# BORDERLINE PERSONALITY DISORDER: ATTITUDES OF PSYCHIATRY AND FAMILY MEDICINE RESIDENTS

**DOI:** 10.1192/j.eurpsy.2023.2050

**Published:** 2023-07-19

**Authors:** B. Olfa, O. Sana, A. Syrine, F. Rim, S. Najeh, G. Imen, C. Nada, B. T. Jihen, Z. Lobna, M. B. Manel, M. Mohamed

**Affiliations:** Psychiatry C Department, Hedi chaker University hospital, sfax, Tunisia

## Abstract

**Introduction:**

Patients with Borderline Personality Disorder (BPD) require a significant amount of time and effort on the part of general practitioners and psychiatrists, resulting in longer visits and complex medical records, with a poor resolution of both physical and mental symptoms.

These patients are likely to express feelings of anger and violence, compared to other patients which makes it difficult to deal with them.

**Objectives:**

To determine attitudes toward patients with BPD among psychiatry and family medicine residents in Tunisia. To understand in addition the challenges that these professionals encounter in their everyday practice

**Methods:**

An online anonymous questionnaires was distributed through social networks to psychiatry residents and family medicine resident.

The attitudes of health professionals towards people with BPD was used to assess clinicians’ attitudes towards people with BPD.

**Results:**

Thirty three clinicians were in the study. A high proportion of respondents (81.8%) were females .The age of the participants ranged from 25 to 34 years, with an average age of 28 years and 3 months (SD = 2.23).In our study 34% reported that they often see patients with borderline personality .The half of the participants (51.5%) reported a feeling being on guard when meeting borderlines while 36.4% were empathetic and neutral. The feeling of anger and frustration was reported with 12.1 % of the participants.

The most frequent cause of these feelings was the difficulty of taking care of patients with BPD.

The majority of the participants (51.5%) reported avoiding working with them. However, only 39.4% reported asking a colleague to replace them in their follow-up.

The results of Attitudes of health professionals towards people with BPD Scale indicate that clinicians generally present a positive attitude towards patient BPD with a mean score 94.94(SD=18.60)

**Conclusions:**

Working with patients with BPD can be challenging. Professionals’ attitude can create obstacles to effective communication and successful treatment.

Therefore, all clinicians should receive more specific training to be able to deal with this condition.

**Disclosure of Interest:**

None Declared

